# GWAS is going to the dogs

**DOI:** 10.1186/gb4166

**Published:** 2014-03-13

**Authors:** Mitchell J Machiela, Stephen J Chanock

**Affiliations:** 1 Division of Cancer Epidemiology and Genetics, National Cancer Institute, Bethesda, MD 20892, USA

## Abstract

Genome-wide association studies in canine models may help locate genomic susceptibility regions that are relevant to human disease.

See related Research: http://genomebiology.com/2014/15/3/R25

## 

Dogs have been called man’s best friend, and for good reasons. For centuries, humans have domesticated and trained canines (*Canis lupus familiaris*) to use both their intelligence and special abilities for a variety of tasks. They serve as watchdogs, guide dogs, sled dogs, herding dogs, sniffing dogs and even Frisbee-catching dogs. Modern dogs are derived from at least two population bottlenecks: the first resulting from domestication from wolves and, very recently, the second from intensive selection deliberately intended to create distinct breeds with particular morphological features (such as height or coat color) and behaviors (such as herding or hunting), frequently suited to particular roles. Consequently, most breeds are based on a small number of founders, which in turn results in limited genetic diversity, characterized by long stretches of linkage disequilibrium [1]. We now know, however, that intense, artificial inbreeding comes at a price: genetic susceptibility to a range of autoimmune diseases, behavioral disorders and cancers, many mimicking aspects of human diseases [2]. Geneticists have capitalized on this high degree of relatedness to investigate the genetics of breed-specific diseases that mimic human disorders.

Initially, the breed structure of dogs enabled geneticists to pinpoint highly penetrant mutations because of limited locus heterogeneity, akin to studying geographically isolated human populations, as successfully pursued in Finland and Iceland. For example, epilepsy [3] and narcolepsy [4] genes were initially mapped in dogs and quickly pursued in humans.

Genome-wide association studies (GWAS) have emerged as an effective approach to agnostically scan genomes with dense microarrays of single nucleotide polymorphisms (SNPs) in search of disease-susceptibility alleles [5]. In particular, GWAS have transformed the discovery of multiple loci associated with risk for complex diseases, such as autoimmune disorders, cancer or diabetes. The GWAS approach is predicated on first finding surrogate markers of susceptibility that are later fine mapped. Eventually, functional variants responsible for disease susceptibility can be characterized, albeit at a slower pace. In humans, GWAS have been fruitful for conclusively identifying thousands of common variants associated with complex diseases. Because of the complex patterns of human evolution, yielding smaller blocks of linkage disequilibrium, large sample sizes (in the thousands) are required for genotyping on arrays containing hundreds of thousands of SNP markers.

Recent artificial selection of dog breeds has simplified matters for canine geneticists. Based on the comparison of whole-genome sequence data for distinct breeds, Lindblad-Toh *et al.*[1] predicted that the lack of genetic heterogeneity in breed structure would simplify GWAS, reducing the number of cases and controls needed by orders of magnitude, along with the number of SNP markers that need to be genotyped. Indeed, many disease-susceptibility alleles have been identified using GWAS, sometimes facilitated by using inter-breed relatedness to quickly pinpoint the disease-associated haplotype [2]. Despite the great success of GWAS, some human diseases remain difficult to study by this approach because of their relatively low prevalence or high degree of genomic complexity. Thus, GWAS in canines can be particularly useful in mapping multigenic traits.

## Unraveling behavioral problems in dogs

In this issue of *Genome Biology*, Tang *et al.*[6] report the combination of GWAS and targeted sequencing to map obsessive-compulsive disorder (OCD) in dogs. The authors [6] cleverly transitioned from GWAS to the judicious use of next-generation sequencing to identify new OCD alleles. OCD is a common and debilitating neuropsychiatric disorder characterized by persistent intrusive thoughts and time-consuming repetitive behaviors. It is the fourth most common psychiatric disorder in humans, with a lifetime prevalence of approximately 2%. Twin studies demonstrate a strong genetic component, and first-degree relatives of an affected individual are at an increased risk of developing disease. A GWAS conducted with 1,465 human OCD cases and 400 family trios did not successfully uncover new disease-susceptibility loci [7], suggesting a highly complex underlying genetic architecture.

Canines also present with naturally occurring OCD, which can manifest as repetition of normal canine behaviors. The authors [6] reanalyzed data from an initial canine GWAS [8]; promising regions of marginal significance were then selected for targeted sequencing in eight OCD dogs and eight controls [6]. In total, 2,291 case-only variants were discovered, of which a subset of 114 were found to be significantly more common in OCD-risk breeds when genotyped in an independent sample. Gene-based analyses revealed that cadherin 2 (*CDH2*), catenin alpha 2 (*CTNNA2*), ataxin 1 (*ATXN1*) and plasma glutamate carboxypeptidase (*PGCP*) harbored the most case-only variants; these initial canine susceptibility loci, which are all reported to have synaptic functions, may also be associated with human OCD.

## Identifying cancer susceptibility alleles in dogs by GWAS

Recently in *Genome Biology*, Karlsson *et al.*[9] reported a pllel, multi-breed GWAS approach to study osteosarcoma, a primary bone malignancy that is also observed in children and adolescents. Osteosarcoma is the most common primary bone malignancy in children and young adults, affecting approximately four per million adolescents in the United States each year, with a high mortality rate in excess of 30%. Peak incidence occurs during the pubertal growth spurt, and established risk factors include tall stature and high birth weight. A recent human GWAS of nearly 1,000 osteosarcoma cases identified regions on 2p25.2 and 6p21.3 that were significantly associated with osteosarcoma risk [10]. One locus, marked by rs7591996 (odds ratio 1.39, 95% confidence interval 1.23 to 1.54, *P* = 1.0 × 10^-8^) at 2p25.2 mapped to an intergenic region, but the second locus, marked by rs1906953 at 6p21.3 (odds ratio 1.57, 95% confidence interval = 1.35 to 1.83, *P* = 8.0 × 10^-9^) mapped to intron 7 of a plausible candidate gene, glutamate receptor metabotropic 4 (*GRM4*). *GRM4* is involved in intracellular signaling and inhibition of the cyclic AMP signaling cascade.

Canine osteosarcoma is clinically similar to human osteosarcoma and has the advantage of being common among certain breeds (such as greyhounds, Rottweilers and Irish wolfhounds). Remarkably, in this study of 309 dogs from three breeds [9], 33 osteosarcoma-associated loci were discovered that can explain perhaps 50% of disease variation within each breed. Interestingly, there was no overlap in regions of association between breeds, although small sample size and fixation of several risk haplotypes may have prevented seeing these associations across breeds. Pathway analyses of human genomic regions syntenic to the canine GWAS loci revealed significant connections related to growth, osteoblast differentiation and proliferation, and tumor suppression. Only a fraction of the genes and pathways reported in the canine GWAS have been previously implicated in osteosarcoma, further demonstrating the power of agnostic GWAS to uncover novel regions of the genome in canine models. Although *GRM4* was not specifically replicated in the canine GWAS, another glutamate receptor gene, *GRIK4*, was significantly associated in greyhounds, and regions near *GRM4* reached fixation in Rottweilers.

## Confirmation of why dogs are man’s best friends

The successful discovery by GWAS of new susceptibility alleles for canine osteosarcoma and OCD exemplifies the value of conducting genetic studies in dogs. It is notable that in these two instances, there is a striking similarity in the disease phenotypes between dogs and humans. One might imagine, therefore, that the canine findings are likely to replicate in humans and eventually translate into new biological insights that could lead to new strategies for diagnosis, prevention, or treatment.

Having already applied next-generation sequencing technologies to solving complex genetic models in dogs, we can expect to see many more disease loci mapped by canine geneticists. Furthermore, because of the artificial inbreeding in dogs, it may be easier to use SNP microarrays to discover disease-associated structural and copy-number variations. When these analytical approaches are optimized in dogs they may, in turn, be applied to human disease mapping. In this regard, our furry friends may teach humans new tricks with old methods.

In summary, genetic insights gained from canine disease models have the potential to accelerate pllel discovery in humans and eventually advance the implementation of precision medicine. GWAS is indeed going to the dogs, but we should be thankful that our loyal and devoted companions can offer such rich opportunities for discovery. Yes, man’s best friend indeed has extended a paw to help humans map complex diseases.

## Abbreviations

GWAS: Genome-wide association study; OCD: Obsessive-compulsive disorder; SNP: Single nucleotide polymorphism.

## Competing interests

The authors declare that they have no competing interests.
